# Forage Species and Nutrition Among Reintroduced Banteng (*Bos javanicus* d'Alton, 1823) in Salakphra Wildlife Sanctuary and Khao Kiew–Khao Chompoo Wildlife Sanctuary, Thailand

**DOI:** 10.1002/ece3.73401

**Published:** 2026-04-01

**Authors:** Wasinee Thepapichaikul, Rattanawat Chaiyarat, Roschong Boonyarittichaikij, Phanwimol Tanhan, Seree Nakbun

**Affiliations:** ^1^ Master of Science Program in Appropriate Technology and Innovation for Environment, Faculty of Environment and Resource Studies Mahidol University Salaya Nakhon Pathom Thailand; ^2^ Wildlife and Plant Research Center, Faculty of Environment and Resource Studies Mahidol University Salaya Nakhon Pathom Thailand; ^3^ Wildlife and Exotic Animal Studies, Faculty of Veterinary Science Mahidol University Salaya Nakhon Pathom Thailand; ^4^ Department of Pharmacology, Faculty of Veterinary Medicine Kasetsart University Bangkhen Bangkok Thailand; ^5^ Department of National Parks, Wildlife and Plant Conservation Khao Nam Phu Nature and Wildlife Education Center Sri Sawat Kanchanaburi Thailand

**Keywords:** Banteng, fecal analysis, forage, niche, nutrition, reintroduction

## Abstract

Dietary management is an important factor affecting the health and survival of critically endangered wildlife—such as banteng recently reintroduced into Salakphra Wildlife Sanctuary (SWS) and Khao Kiew–Khao Chompoo Wildlife Sanctuary (KKKC). This study investigates the diversity and nutritional composition of forage consumed by different reintroduced banteng practices in these two protected areas. The research was conducted between November 2023 and October 2024 using fecal analysis and mineral composition assessments. A total of 59 forage species (51 consumed in the wet season, 35 in the dry season) were identified in banteng feces in SWS. These 59 species consisted of 29 monocotyledons (49.15%) and 30 dicotyledons (50.85%). The most preferred species found in banteng diets in the dry and wet seasons were *Cyrtococcum* sp. and *Dendrolobium triangulare* (Retz.) Schindl., respectively. A total of 36 species (28 in the wet season, 22 in the dry season) were found in banteng feces in KKKC. These 36 forage species consisted of 27 monocotyledons (75%) and 9 dicotyledons (25%). The most preferred diets in the dry and wet season were *Panicum maximum* Jacq. and *Imperata cylindrica* (L.), respectively. The SWS showed a significant seasonal difference (*p* = 0.015) which was also higher than in KKKC in both seasons. The highest‐preferred forages in each season were then collected to measure their mineral content. The food plants from both areas contained adequate N, P, S, K, Ca, Mg, Na, Cu, Fe, Mn, and Zn—all of which contributed to banteng nutritional requirements. The results also indicated that reintroduced banteng exhibit dietary flexibility and adaptability to different natural habitats.

## Graphical abstract

Dietary management is an important factor affecting the health and survival of critically endangered wildlife. The natural habitat showed a significant seasonal difference which was also higher than in introduced habitat. Reintroduced banteng exhibit dietary flexibility and adaptability to different natural habitats.

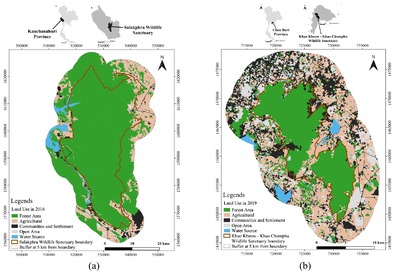

## 1 Introduction

Over the past decades, captive breeding, reintroduction, and translocation programs have emerged as increasingly significant tools in conservation practice (Sankar et al. 2013). The reintroduction programs have also been adapted for several different wildlife species. Successful reintroductions of mammals include: Arabian oryx ( *Oryx leucoryx* Pallas, 1777) in Oman (Fitter 1990), bison ( *Bison bison* Linnaeus, 1758) in Canada (Kay and White 2001), red deer ( *Cervus elaphus* ) in Portugal (Valente et al. 2017), and European bison ( *Bison bonasus* Linnaeus, 1758) in central‐eastern portions of the Europe Union (Lord et al. 2019), etc.

Banteng ( *Bos javanicus* d'Alton, 1823) belong to the family Bovidae and are categorized as a critically endangered species by the International Union for the Conservation of Nature (IUCN) and are red listed (Timmins et al. 2019). The global population has seen a decrease in excess of 50% caused by poaching, habitat loss, degradation, anthropogenic disturbance, and disease transmitted by domestic cattle ( *B. taurus* and *B. indicus* ) (Gardner et al. 2016). Banteng are distributed mainly in Southeast Asia, particularly in Cambodia, Indonesia (Java, Kalimantan), Lao PDR, Malaysia (Sabah), Myanmar, Thailand, and Vietnam. Banteng play an important ecological role as prey for keystone predators, such as tigers ( *Panthera tigris* linnaeus, 1758) (Simcharoen et al. 2018). In Thailand, two releasing sites have been established for banteng at Khao Kiew–Khao Chompoo Wildlife Sanctuary (KKKC) in Chonburi Province (Chaiyarat et al. 2018) and Salakphra Wildlife Sanctuary (SWS) in Kanchanaburi Province (Chaiyarat et al. 2019). The differences in release strategies between the two sites. In KKKC, banteng escaped from Khao Keio Open Zoo (Chaiyarat et al. 2018) into KKKC that was not their original habitat, while in SWS was prepared from pre‐release preparation, and release methods including feasibility assessments in the previous habitat before they were locally extinct (Chaiyarat et al. 2019). These influenced the success of the reintroduction programs in these two areas. To date, both populations have continued to survive in the areas (Kongsurakan et al. 2020).

Monitoring is a critical component of wildlife reintroduction programs. It provides valuable data for assessing the success of efforts and helps inform future management decisions. The habits and nutrition of banteng diets post‐release also reveal their adaptive ability to survive in a given new habitat. It is crucial to analyze the population's health, and a key way of doing that is by investigating the forage they consume, as well as its ability to provide essential macro and microminerals. Banteng are browsers rather than grazers. A direct observation from released banteng in KKKC found 23 plant species identified, where only five species, predominantly from the family Poaceae, were found (Chaiyarat et al. 2018). While reintroduced banteng in SWS consumed a total of 24 plant species (Chaiyarat et al. 2020). The knowledge of adaptive feeding behavior is essential for the long‐term conservation of reintroduced banteng populations. The aim of the research was to study food habits and nutrition compositions in forage species of banteng after releasing into natural habitats for ensuring the sustainability of a reintroduction program. These outcomes will enhance the prospects of increasing banteng numbers in the wild and ultimately contribute to the restoration of the ecosystem.

## 2 Materials and Methods

### 2.1 Study Areas

**FIGURE 1:**
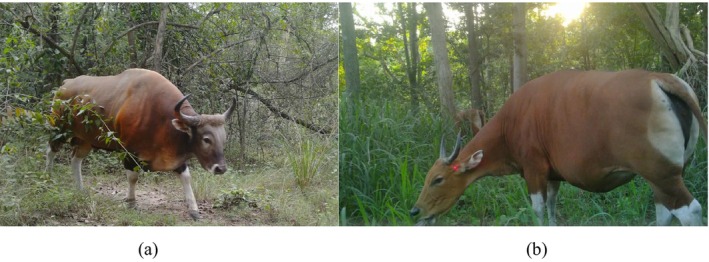
Reintroduced banteng in (a) Salakphra Wildlife Sanctuary and (b) Khao Kiew–Khao Chomphu Wildlife Sanctuary, Thailand (Photos credit: Rattanawat Chaiyarat).

**FIGURE 2:**
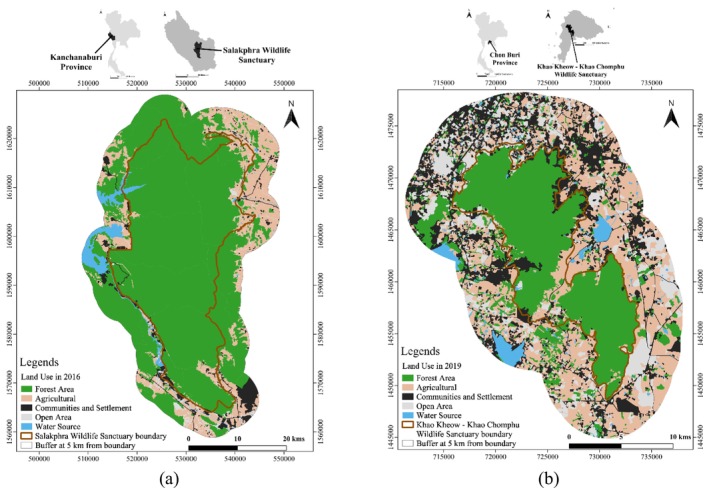
Banteng reintroduction areas in (a) Salakphra Wildlife Sanctuary and (b) Khao Kiew–Khao Chomphu Wildlife Sanctuary, Thailand.

The study was conducted in two protected areas where banteng (Figure 1) were released in Thailand: SWS is located in the southern part of the western forest complex at 14°8′37.09″ N and 99°20′33.51″ E in Kanchanaburi Province with a total area of 860 km^2^ (Figure 2a). The sanctuary is covered mainly by 60% mixed deciduous forest, 30% dry dipterocarp forest and 10% disturbed areas. The *Lagerstroemia tomentosa* C. Presl, *Terminalia alata* Heyne ex Roth, *T. triptera* Stapf, *T. bellirica* (Gaertn.) Roxb. and *Afzelia xylocarpa* (Kurz) Craib are the dominant plant species (Salakphra Wildlife Sanctuary 2011). The variety of known wildlife includes the Asian elephant ( *Elephas maximus* ), tiger (*Pantera tigris*), gaur, banteng, several species of deer, other small mammals and birds (Department of National Parks, Wildlife and Plant Conservation [DNP] 2023a). In December 2015, the first banteng group was released into the area. The program has been continuously implemented [16]. In 2024, five events were successfully carried out, resulting in the release of 16 bantengs (10 males and 6 females) into the wild. At least 26 new calves have been recorded within the herd since (Table S1).

Khao Kiew–Khao Chompoo Wildlife Sanctuary is located in Chonburi Province, at 13°14′45″ N 101°4′15″ E. The total area encompasses 144.7 km^2^ (Figure 2b). The plant community is composed of 37.5% mixed deciduous forest, 51.6% dry evergreen forest, 6.3% agricultural area, 2.1% grassland, 2.2% secondary forest and 0.3% water sources. The dominant plant species included *Streblus asper* Lour., *Pterocarpus macrocarpus* Kurz, *Diospyros rhodocalyx* Kurz, *Antidesma bunius* Spreng., and *Wrightia arborea* (Dennst.) Mabb. The known wildlife in the area includes 25 species of mammals, 106 species of birds, 18 species of amphibians and 36 species of reptiles (DNP 2023b). In 1988, 13 captive bantengs at the Khao Kiew Open Zoo were unintentionally released into the adjacent forest of the KKKC (Prakobphon 1988). Since their release, the population has increased from the initial 13 individuals to a minimum of 34 individuals (Chaiyarat et al. 2018) (Table S1).

### 2.2 Fecal Sampling

All fecal and forage samples were collected from SWS and KKKC between November 2023 and October 2024. The sampling period was divided into two seasons: the dry season (November to April) and the wet season (May to October). Feces were identified based on its size and shape, and identification was confirmed using camera traps installed at frequently used defecation sites. Thirty fecal samples were collected in each area per season in clean zip‐lock plastic bags, at a minimum weight of 100 g. Samples were obtained only from the middle of fresh feces to avoid contamination by plant or soil material. The samples were stored at a refrigerated temperature until used for further analysis (Putman 1984).

### 2.3 Fecal Analysis

Fecal analysis was conducted following the methodology of Anthony and Smith (1974). The effectiveness and reliability of fecal analysis are influenced by several factors, the characteristics of its diet, and the diversity of available forage plants. Certain food items, such as flowers, tubers, seeds, or acorns, are often highly digestible and may lack distinctive epidermal structures; a highly diverse diet would make the technique more time consuming and may increase the misidentification due to overlapping microscopic characteristics among species (Anthony and Smith 1974). A teaspoon of feces was cleaned by washing it with tap water and filtering out any dirt. It was boiled in 20 mL of tap water for 30 min. A solution of 70% HNO_3_ was added and boiled for another 10 min. Five drops of formalin were added to preserve the extracts (Morrison 2008). A light microscope with a 10× and 40× lens was then used to examine ten pieces of forage from each sample. Photos of all samples were taken and compared for reference. The plant field samples of the available plant species from each season were collected and prepared as reference slides of epidermal cells of plant species. Documentation from Prayurasiddhi et al. (1999) and Chaiyarat et al. (2020) were also used for identification. All fecal plant fragments were noted first as monocotyledon or dicotyledon. Further fecal analysis was undertaken to confirm the given banteng's forage species consumed and to determine species' relative abundance (RA) and frequency (RF) in the diet. The calculated using the following equation:

$$\mathrm{RA}=\left(\frac{\text{Total number of individuals of specie}\;i}{\text{Total number of individuals of}\;\mathrm{all}\;\text{species}}\right)\times 100$$

$$\mathrm{RF}=\left(\frac{\text{Total number of feces with specie}\;i\;\text{observed}}{\text{Total number of fecal sample}}\right)\times 100$$

### 2.4 Ecological Niche Index

Niche breadth for each season within the study area was assessed. The Levin's measure of niche breadth calculation was using the equation as follows (Krebs 1999):

$$B=\frac{1}{\Sigma p_{i}^{2}}$$

Standardized niche breadth equation

$$B^{\prime }=\frac{B-1}{n-1}$$

where *B*, Levin's measure of niche breadth; *B*′, Standardized niche breadth; *p*_*i*_, Proportion of individuals found in or using resource state; *i*, *n*, number of possible resource state.

Seasonal niche overlap was evaluated using Pianka's index of niche overlap, among area and season. Pianka's measure of niche overlap was calculated using the equation given below (Pianka 1973).

$$O_{\mathrm{jk}}=\frac{\sum_{i=1}^{n}p_{\mathrm{ij}}\cdot p_{\mathrm{ik}}}{\sqrt{\left(\sum_{i=1}^{n}p_{\mathrm{ij}}^{2}\right)\cdot \left(\sum_{i=1}^{n}p_{\mathrm{ik}}^{2}\right)}}$$

where *O*_*jk*_, Pianka's index of niche overlap between species *j* and *k*; *p*_*ij*_, Proportion of resource *i* used by species *j*; *p*_*ik*_, Proportion of resource *i* used by species *k*; *n*, Total number of resource categories.

### 2.5 Forage Nutritional Analysis

Ten samples of plant species identified as the most frequently consumed (based on RA and RF) were collected from banteng feeding areas. Information was then sought on the chemical composition of macro and microminerals therein. The forage samples were collected from both study sites at the end of the wet and dry seasons. The identification of selected plants was confirmed with the help of forest rangers and the Wildlife and Plant Research Center, Faculty of Environment and Resource Studies, Mahidol University.

Mineral composition was analyzed in accordance with the standards established by the Food and Agriculture Organization of the United Nations (FAO 2009). Levels of Nitrogen (N) were determined using the Micro‐Kjeldahl method, Phosphorus (P) by Acid Digestion and Spectroscopy, and Sulfur (S) by Acid Digestion and Turbidimetric. Meanwhile, Potassium (K), Calcium (Ca), Iron (Fe), Magnesium (Mg), Manganese (Mn), Sodium (Na), Copper (Cu), and Zinc (Zn) were determined by using Acid Digestion and Atomic Spectroscopy. All sample testing was performed at the Soil Plant and Agricultural Material Testing and Research Unit, Central Laboratory and Greenhouse Complex, Kasetsart University.

### 2.6 Statistical Analysis

Banteng were divided into four groups correlated to sampling from the two areas by season. Forage species in feces were counted by seasons and areas, and then were compared using ANNOVA and Tukey's test. Statistical significance here was considered to be at *p* ≤ 0.05. All statistics were analyzed by using R Studio version 2024.12.0.

The mineral content of distinguished forage species was then analyzed using multivariate statistical methods and cluster analysis, and by applying Non‐metric Multidimensional Scaling (NMDS) (Sun et al. 2025). This approach was employed to examine the relationships between plant species and their mineral compositions.

## 3 Results

### 3.1 Forage Diversity, Food Habit and Preference

A total of 120 fecal samples were collected, including 30 samples from SWS in both the dry and wet seasons, and 30 samples were similarly collected from KKKC during each of the two seasons. A total of 82 forage species were then identified in the banteng feces across two study areas, composed of 44 monocotyledon and 38 dicotyledon species, respectively. Of the 82 forage species consumed by banteng, 59 species were recorded in the SWS area and 36 species in the KKKC area, with 13 species being common to both study sites (Table 1).

**TABLE 1:** The Relative abundance (RA) and Relative frequency (RF) in the dry (Dry) and the wet (Wet) seasons of forage species found in banteng feces from two reintroduction areas: Salakphra Wildlife Sanctuary (SWS) and Khao Kiew–Khao Chompoo Wildlife Sanctuary (KKKC) between November 2023–October 2024.

Family	Scientific name	SWS				KKKC			
		RA (%)		RF (%)		RA (%)		RF (%)	
		Dry	Wet	Dry	Wet	Dry	Wet	Dry	Wet
**Monocotyledons**									
Araceae	*Rhaphidophora* sp.	3.3	2.3	33.3	23.3	—	1	—	6.7
Arecaceae	*Cocos nucifera* L.	—	—	—	—	0.3	1	3.3	6.7
Cyperaceae	*Cyperus brevifolius* (Rottb) Hassk.	—	2	—	20	—	—	—	—
	*Cyperus haspen* L.	0.7	—	6.7	—	—	1.3	—	13.3
	*Fimbristylis insignis* Thwaites.	0.3	3.7	3.3	26.7	4.3	0.3	30	3.3
Poaceae	*Apluda mutica* L.	5.7	0.7	36.7	6.7	—	0.3	—	3.3
	*Aristida balansae* Henrard.	—	2	—	16.7	0.3	—	3.3	—
	*Arundinella rupestris* A.Camus	1	6.3	10	40	—	—	—	—
	*Axonopus compressus* (Sw.) P.Beauv.		1.7	—	16.7	0.3	—	3.3	—
	*Bambusa bambos* (L.) Voss	12.3	5	53.3	30	—	—	—	—
	*Brachiaria mutica* (Forssk.) Stapf	—	—	—	—	9	17.7	53.3	70
	*Chloris barbata* Sw.	0.3	6.7	3.3	33.3	0.7	1.7	6.7	10
	*Cymbopogon nardus* (L.) Rendle	—	0.3	—	3.3	—	—	—	—
	*Cyrtococcum acresscens* (Trin.) Stapf	3	—	23.3	—	—	—	—	—
	*Cyrtococcum* sp.	13.7	13.3	53.3	43.3	—	0.3	—	3.3
	*Dendrocalamus strictus* (Roxb.) Nees	2	7	16.7	43.3	—	—	—	—
	*Digitaria ciliaris* (Retz.) Koeler. var. *chrysoblephara* (Figari & De Notaris) R. R. Stewart	5.3	4	33.3	33.3	—	—	—	—
	*Hyparrhenia rufa* (Nees) Stapf	—	0.3	—	3.3	0.7	2.3	6.7	20
	*Imperata cylindrica* (L.) Raeusch.	—	—	—	—	27	24.3	80	86.7
	*Ophiuros* sp.	—	—	—	—	0.3	4.7	3.3	30
	*Panicum maximum* Jacq.	—	—	—	—	38.7	25.7	96.7	80
	*Panicum repens* L.	5.7	5.7	33.3	33.3	—	—	—	—
	*Saccharum arundinaceum* Retz.	—	—	—	—	0.7	0.7	6.7	6.7
	*Saccharum spontaneum* L.	—	—	—	—	6.3	8.3	43.3	33.3
	*Saccharum* sp.	—	—	—	—	3.7	3	16.7	20
	*Sorghum nitidum* (Vahl) Pers.	—	—	—	—	—	0.3	—	3.3
	*Thyrsostachys siamensis* Gamble	6.7	1.3	30	10	3.3	—	26.7	—
Zingiberaceae	*Zingiber* sp.	1.3	2	13.3	20	—	0.3	—	3.3
N/A	UN monocot 1	—	2	—	16.7	—	—	—	—
	UN monocot 2	1	0.3	10	3.3	—	0.3	—	3.3
	UN monocot 3	0.3	1.7	3.3	6.7	—	—	—	—
	UN monocot 4	0.3	0.7	3.3	6.7	—	—	—	—
	UN monocot 5	0.3	—	3.3	—	—	—	—	—
	UN monocot 6	—	0.3	—	3.3	—	—	—	—
	UN monocot 7	—	0.3	—	3.3	—	—	—	—
	UN monocot 8	—	0.3	—	3.3	—	—	—	—
	UN monocot 9	—	0.3	—	3.3	—	—	—	—
	UN monocot 10	—	0.3	—	3.3	—	—	—	—
	UN monocot 11	—	—	—	—	1	0.3	10	3.3
	UN monocot 12	—	—	—	—	—	0.7	—	6.7
	UN monocot 13	—	—	—	—	—	0.3	—	3.3
	UN monocot 14	—	—	—	—	0.7	0.3	6.7	3.3
	UN monocot 15	—	—	—	—	0.7	—	6.7	—
**Dicotyledons**									
Asteraceae	*Mikania cordata* (Burm.f.) B.L.Rob.	—	—	—	—	—	0.7	—	3.3
Burseraceae	*Garuga pinnata* Roxb.	—	1.3	—	6.7	—	—	—	—
Capparaceae	*Maerua siamensis* (Kurz) Pax.	—	0.3	—	3.3	—	—	—	—
Curcubitaceae	*Trichosanthes cucumerina* L.	—	1	—	10	—	—	—	—
Dioscoreaceae	*Dioscorea hispida* Dennst.	—	—	—	—	0.3	—	3.3	—
	*Dioscorea membranacea* Pierre ex Prain & Burkill	—	—	—	—	—	0.7	—	6.7
Ebenaceae	*Diospyros rhodocalyx* Kurz	—	0.3	—	6.7	—	—	—	—
Fabaceae	*Acacia harmandiana* (Pierre) Gagnep.	—	0.3	—	3.3	—	—	—	—
	*Albizia myriophylla* Benth.	—	—	—	—	—	0.7	—	6.7
	*Bauhinia saccocalyx* Pierre	4.7	1	26.7	10	—	—	—	—
	*Bauhinia scandens* L.	11.3	3.7	50	23.3	—	—	—	—
	*Caesalpinia sappan* L.	3.3	1.3	20	10	—	—	—	—
	*Dalbergia cultrata* Graham ex Benth.	3.3	—	26.7	—	—	—	—	—
	*Dendrolobium triangulare* (Retz.) Schindl.	2.3	7.3	16.7	56.7	—	—	—	—
	*Millettia brandisiana* Kurz	4	2.7	26.7	26.7	—	—	—	—
Malvaceae	*Abutilon indicum* (L.) Sweet	0.3	0.3	3.3	3.3	—	—	—	—
Moraceae	*Streblus asper* Lour.	0.3	1	3.3	10	0.3	2	3.3	13.3
Rhamnaceae	*Ziziphus cambodiana* Pierre.	0.3	1	3.3	6.7	—	—	—	—
	*Ziziphus oenoplia* (L.) Mill. var. *oenoplia*	4.3	2.7	26.7	20	—	—	—	—
Phyllanthaceae	*Antidesma bunius* (L.) Spreng.	—	0.3	—	3.3	—	—	—	—
	*Phyllanthus reticulatus* Poir.	—	0.7	—	6.7	—	—	—	—
Verbenaceae	*Lantana camara* L.	0.3	—	3.3	—	—	—	—	—
N/A	UN dicot 1	0.3	—	3.3	—	—	—	—	—
	UN dicot 2	0.3	—	3.3	—	—	—	—	—
	UN dicot 3	0.3	0.3	3.3	3.3	—	—	—	—
	UN dicot 4	0.3	—	3.3	—	—	—	—	—
	UN dicot 5	0.3	0.3	3.3	3.3	—	—	—	—
	UN dicot 6	0.3	0.3	3.3	3.3	—	—	—	—
	UN dicot 7	—	0.3	—	3.3	—	—	—	—
	UN dicot 8	—	0.7	—	6.7	—	—	—	—
	UN dicot 9	—	0.7	—	6.7	—	—	—	—
	UN dicot 10	—	0.3	—	3.3	—	—	—	—
	UN dicot 11	—	0.3	—	3.3	—	—	—	—
	UN dicot 12	—	0.7	—	6.7	—	—	—	—
	UN dicot 13	—	—	—	—	0.7	—	3.3	—
	UN dicot 14	—	—	—	—	—	0.3	—	3.3
	UN dicot 15	—	—	—	—	0.3	—	3.3	—
	UN dicot 16	—	—	—	—	—	0.3	—	3.3

Abbreviation: UN, unknown.

A total of 59 forage species were noted in SWS. Of these, 51 species were consumed during the wet season, 35 species during the dry season, and 27 species were shared between both seasons. These plant species included 29 species of monocotyledon (49.2%) and 30 species of dicotyledon (50.9%). Of the 35 species noted during the dry season, 18 were monocotyledon (51.4%) and 17 were dicotyledon (48.6%). Of the 51 found in the wet season, 26 were monocotyledons (51%) and 25 were dicotyledons (49%), while a total of 27 species were found throughout the year.

In KKKC, banteng consumed a total of 36 plant species. Of these, 28 species were consumed during the wet season, 22 species in the dry season, and 14 species were identified across all seasons. Among the 36 species were 27 monocotyledons (75%) and 9 dicotyledons (25%). In the dry season, samples contained 22 plant species. These included 18 monocotyledons (81.8%) and 4 dicotyledons (18.2%). A total of 28 species were in turn found in the wet season. These included 22 monocotyledons (78.6%) and 6 dicotyledons (21.4%). A total of 14 species were found in both seasons in the KKKC (Table 1).

Banteng preferences for forage food species were assessed by integration of the RA and RF of each forage species. The highest preference ratings of all samples in SWS from both seasons were *Cyrtococcum* sp. followed by *Bambusa bambos* (L.) Voss, *Bauhinia scandens* L., *Dendrolobium triangulare* (Retz.) Schindl. and *Panicum repens* L. During the dry season, *Cyrtococcum* sp. showed the highest preference followed by *Bambusa bambos* (L.) Voss, *Bauhinia scandens* L., *Apluda mutica* L. and *Panicum repens* L., respectively. In the wet season, the highest preferences noted were *Dendrolobium triangulare* (Retz.) Schindl. followed by *Cyrtococcum* sp., *Dendrocalamus strictus* (Roxb.) Nees, *Arundinella rupestris* A.Camus and *Chloris barbata* Sw., respectively.

The species with the highest preference from KKKC in all samples from both seasons were *Panicum maximum* Jacq., followed by *Imperata cylindrica* (L.) Raeusch., *Brachiaria mutica* (Forssk.) Stapf., *Saccharum spontaneum* L. and *Saccharum* sp. In the dry season, the species with the highest preference were *Panicum maximum* Jacq., followed by *Imperata cylindrica* (L.) Raeusch., *Brachiaria mutica* (Forssk.) Stapf., *Saccharum spontaneum* L. and *Fimbristylis insignis* Thwaites. During the wet season, the highest preferences were noted as *Imperata cylindrica* (L.) Raeusch., followed by *Panicum maximum* Jacq., *Brachiaria mutica* (Forssk.) Stapf., *Saccharum spontaneum* L. and *Ophiuros* sp. It was observed that all dicotyledon species showed low values in both seasons in KKKC.

The calculated average number of forage species in fecal samples for SWS in the wet season was 7 ± 1.5 species; for SWS in the dry season 6 ± 1.2 species; for KKKC in the wet season 4.5 ± 1.2 species; and for KKKC in the dry season 4.2 ± 1.1 species. A highly significant difference was observed between the two study areas (*p* = 3.4 × 10^−15^), and between the dry and wet seasons (*p* = 0.005). In contrast, interactions between areas and seasons did not show as statistically significant (*p* = 0.152). In both areas, the wet season generally had a higher species count than the dry season, and the SWS area showed a higher forage diversity than KKKC in both seasons.

**FIGURE 3:**
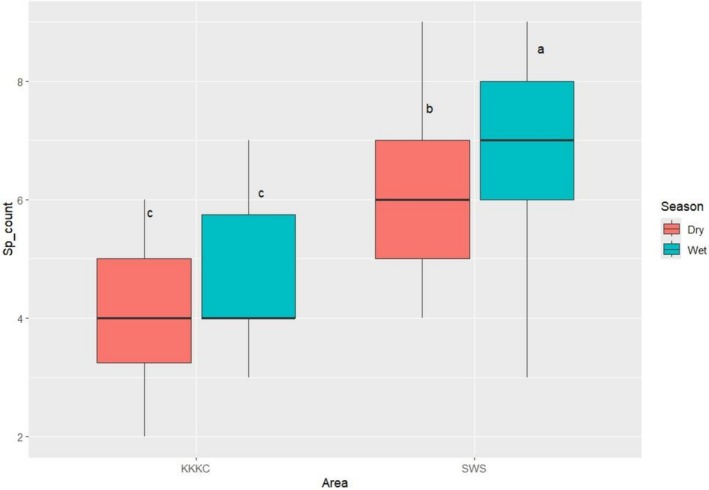
Average forage species in banteng feces in two wildlife sanctuaries between November 2023 and October 2024. KKKC, Khao Kiew–Khao Chompoo Wildlife Sanctuary; SWS, Salakphra Wildlife Sanctuary. Results deemed not significantly different at *p* ≤ 0.05.

According to the ANOVA test, KKKC showed no significant seasonal difference in the average number of forage species in fecal samples (*p* = 0.739). However, significant differences were observed when comparing the two areas in both the dry and wet seasons. The SWS area in the dry season had significantly higher species counts than KKKC in the dry season (*p* < 0.001) and the wet season (*p* < 0.001). Furthermore, the SWS in the wet season was significantly higher than KKKC during both seasons (*p* < 0.001). Within SWS, the species count was significantly higher in the wet season compared to the dry season (*p* = 0.015) (Figure 3).

The results of Tukey's HSD post hoc test revealed significant differences in the average number of forage species found in fecal samples across sites and seasons. The highest species count was observed in SWS during the wet season (group “a”) and was significantly different from all other groups. SWS in the dry season (group “b”) had a moderately high species count and was significantly lower than SWS during the wet season (group “a”) but also higher than KKKC in both the dry and the wet seasons (group “c”), which in turn showed the lowest counts and were not significantly different from each other.

### 3.2 Forage Mineral Composition

A total of 11 mineral elements were analyzed across 40 plant samples (10 species per site in each season). The forage of SWS during the dry season included samples noted to contain high levels of N, S, K, Na, Cu, Fe, and Zn—especially in *Bambusa bambos* (L.) Voss and *Cyrtococcum* sp. During the wet season, the *Dendrolobium triangulare* (Retz.) Schindl. was also one of the most preferred forage species and contained high levels of N and Zn. The *Bambusa bambos* (L.) Voss and *Dendrolobium triangulare* (Retz.) Schindl., meanwhile, were both among the most preferred in the dry and the wet seasons and were found to contain the highest concentration of Zn when compared to other plants during their respective seasons. The second species of preference across both seasons was *Cyrtococcum* sp., which also showed high levels of K, Cu, and Fe (Table S2).

In KKKC, the *Panicum maximum* Jacq. and *Imperata cylindrica* (L.) Raeusch. were the top two most‐consumed species with moderate to low levels of minerals. The other species with lower preference, such as *Brachiaria mutica* (Forssk.) Stapf, were found to contain higher levels of minerals than all other food plants in the area during both seasons (particularly N, S, K, Fe, and Zn). However, in the wet season, *Panicum maximum* Jacq. was found to have a high level of Cu, while *Brachiaria mutica* (Forssk.) Stapf recorded the highest level of N among all food plants. Furthermore, during the dry season, an unidentified monocotyledon species (number 13) recorded the highest concentration of Cu for that season. It was also the least consumed species. Similarly, *Dioscorea hispida* Dennst. contained the highest seasonal levels of N, P, Ca, and Fe among the plants in the wet season, while *Chloris barbata* Sw. was the least consumed species even though it contained the highest concentration of Na (Table S2).

### 3.3 Levin's Niche Breadth Index

Through the sampling from the two areas across seasons, Levin's Niche Breadth (B) showed that SWS during the wet season had the highest adaptability, with an index value of 19.2, followed by SWS during the dry season at 13.9. At the same time, KKKC in the wet and the dry seasons had values of 5.9 and 4.2, respectively. Furthermore, within the same area, the index for the wet season was higher than the dry season in both areas.

The Standardized Levins' Index (BA) revealed that SWS during the wet season had the highest adaptability with an index value of 0.225, followed by SWS in the dry season at 0.159—whereas KKKC in the wet and the dry seasons had values of 0.061 and 0.039 respectively. A low Levin's standardized niche breadth value, close to 0, indicates a tendency for banteng to consume specific food types preferentially. In the KKKC, the most frequently consumed plant species were *Panicum maximum* Jacq. and *Imperata cylindrica* (L.) Raeusch. In contrast, banteng in the SWS exhibited a more generalist feeding behavior when compared to those in KKKC.

### 3.4 Pianka's Niche Overlap Index

**FIGURE 4:**
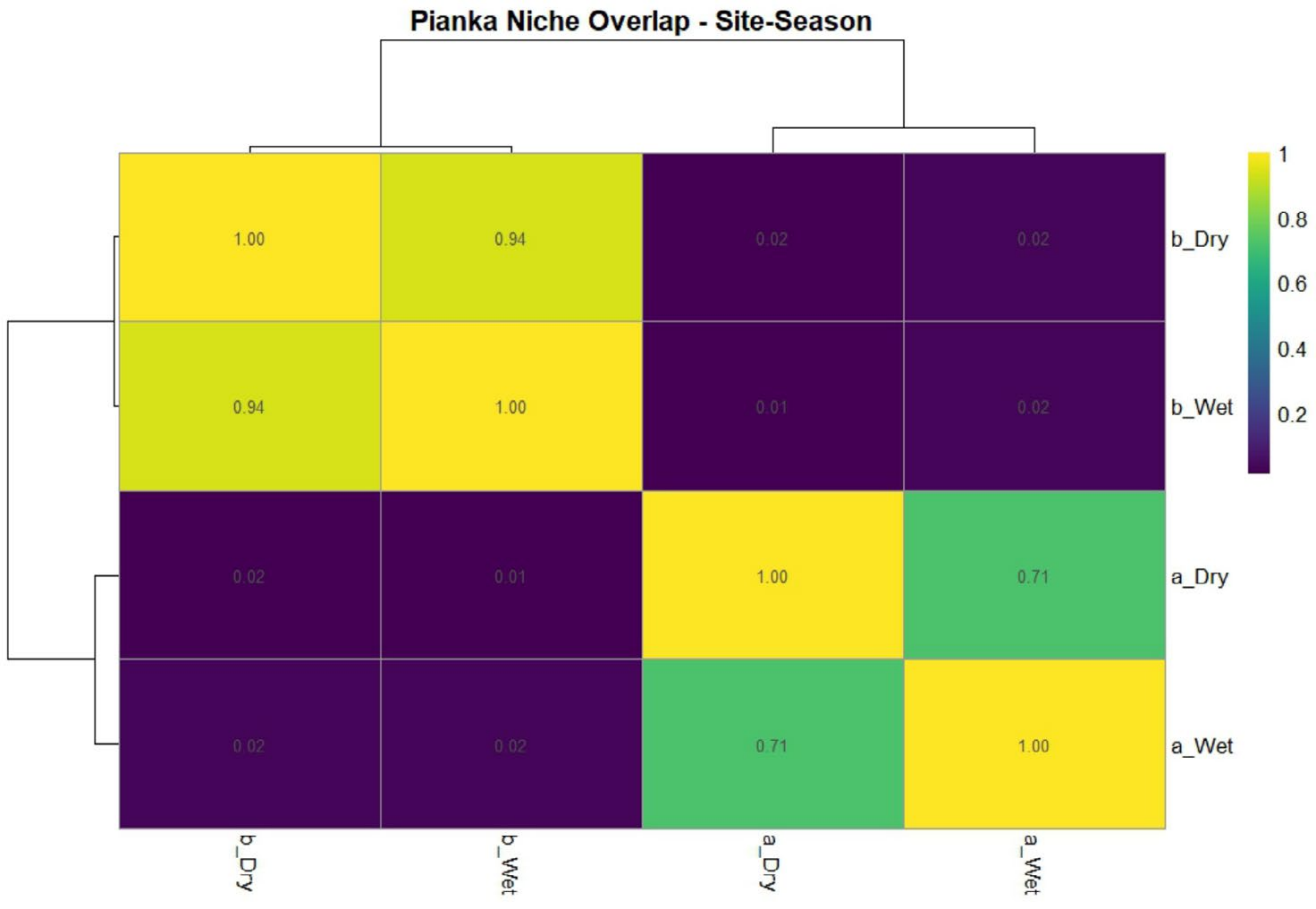
Pianka's niche overlap index of forage species found in banteng feces between two habitat areas from November 2023 to October 2024. a, Salakphra Wildlife Sanctuary; b, Khao Kiew–Khao Chompoo Wildlife Sanctuary.

Seasonal niche overlap was calculated based on Pianka's Niche Overlap Index. The highest overlap was observed between the dry and wet seasons in KKKC at 0.94, followed by the duration between the dry and wet seasons in SWS at 0.71. A value close to 1 indicates that the consumed plant species shows little to no difference between seasons. The lowest overlap was found between the dry season in SWS and the wet season in KKKC at 0.01 (Figure 4). The relatively low niche overlap values indicate seasonal differences in diet composition within each wildlife sanctuary (Figure 4).

### 3.5 The NMDS Ordination

**FIGURE 5:**
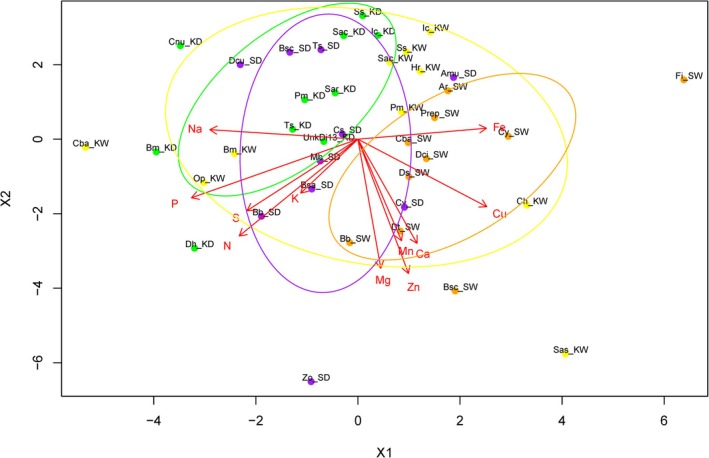
Variation in forage species, associated with mineral composition in Khao Kiew–Khao Chompoo Wildlife Sanctuary and Salakphra Wildlife Sanctuary during the wet and the dry seasons. Points on the plot represent individual forage species samples, color‐coded by site and season, while the overlaid vectors represent gradients of mineral concentrations. Ca, calcium; Cu, copper; Fe, iron; K, potassium; Mg, magnesium; Mn, manganese; *N*, nitrogen; Na, sodium; *P*, phosphorus; S, sulfur; Zn, zinc. Other abbreviations in the chart include: Amu, *Apluda mutica* L.; Ar, Arundinella rupestris A.Camus; Bb, *Bambusa bambos* (L.) Voss; Bm, *Brachiaria mutica* (Forssk.) Stapf; Bsa, Bauhinia saccocalyx Pierre; Bsc, Bauhinia scandens L.; Cba, *Chloris barbata* Sw.; Ch, Cyperus haspen L.; Cnu, *Cocos nucifera* L.; Cs, *Caesalpinia sappan* L.; Cy, Cyrtococcum sp.; Dci, *Digitaria ciliaris* (Retz.) Koeler. var. chrysoblephara (Figari & De Notaris) R. R. Stewart; Dcu, Dalbergia cultrata Graham ex Benth.; Dh, *Dioscorea hispida* Dennst.; Ds, *Dendrocalamus strictus* (Roxb.) Nees; Dt, Dendrolobium triangulare (Retz.) Schindl.; Fi, Fimbristylis insignis Thwaites.; Hr, *Hyparrhenia rufa* (Nees) Stapf; Ic, *Imperata cylindrica* (L.) Raeusch.; KD, Khao Kiew–Khao Chompoo Wildlife Sanctuary in the dry season (green dot); KW, Khao Kiew–Khao Chompoo Wildlife Sanctuary in the wet season (yellow dot); Mb, Millettia brandisiana Kurz; Op, Ophiuros sp.; Pm, *Panicum maximum* Jacq.; Prep, *Panicum repens* L.; Sac, Saccharum sp.; Sar, *Saccharum arundinaceum* Retz.; Sas, Streblus asper Lour.; SD, Salakphra Wildlife Sanctuary in the dry season (purple dot); SW, Salakphra Wildlife Sanctuary in the wet season (orange dot); Ss, *Saccharum spontaneum* L.; Ts, Thyrsostachys siamensis Gamble; UnkDi13, Unknow dicotyledon species number 13; Zo, Ziziphus oenoplia (L.) Mill. var. oenoplia.

The NMDS ordination analysis revealed differences in the mineral composition of forage species across sites and seasons. The result was classified into four distinct groups (Figure 5). amples from KKKC in the dry season were more widely dispersed but generally remained aligned with vectors Na, P, S, and N suggesting a relatively higher presence of these elements during this period. Despite comparatively low overall mineral concentrations, several species within this cluster were highly preferred by banteng, including *Panicum maximum* Jacq. and *Imperata cylindrica* (L.) Raeusch., indicating that dietary preference was consistent despite their limited mineral content. In contrast, samples formed a distinct cluster associated with the SWS during the dry season. This cluster exhibited variable mineral profiles but was characterized by lower preference levels. Their separation from other clusters suggests site‐specific mineral composition patterns that may be influencing forage selection. The cluster representing SWS during the wet season aligned strongly with vectors for Fe, Cu, Ca, Mn, Zn, and Mg, suggesting an association with higher seasonal concentrations of these minerals in forage species.

Notable outliers included *Chloris barbata* Sw. (Cba_KW), *Ziziphus oenoplia* (L.) Mill. var. *oenoplia* (Zo_SD), *Streblus asper* Lour. (Sas_KW) and *Fimbristylis insignis* Thwaites. (Fi_SW), all of which possibly represent rare or unique mineral profiles. Forage species collected during the wet season tend to group toward the right side of the ordination space and are strongly associated with higher levels of micronutrients such as Fe, Cu, and Zn. This pattern suggests that during the wet season, banteng have access to forage species with richer trace mineral content, possibly due to increased soil moisture and plant metabolic activity enhancing mineral uptake. In contrast, species collected during the dry season are generally dispersed toward the left and lower quadrants of the plot and are more closely aligned with macronutrients such as Na, P, and S. This shift may reflect seasonal depletion of micronutrients or changes in plant phenology that affect mineral availability (Figure 5).

## 4 Discussion

In total, 82 forage species were identified within the diets of reintroduced banteng across the two study areas. Each forage species has its own distinct cuticular characteristics and can be identified microscopically in fecal samples of herbivores (Stewart 1970), indicating that fecal analysis is a reliable and practical technique for investigating the dietary ecology of ungulates. Fecal analysis provides a non‐invasive method; however, its limitations depend on the diversity of available plants prepared in reference slides. For instance, some parts of plant food tissue, such as flowers, sticks, or fruits, cannot be identified in fecal samples (Anthony and Smith 1974).

In the SWS, high preference foods aligned mostly with the forest types and cover, which are composed largely of mixed deciduous and dry dipterocarp forests. All highly consumed plant species were commonly found in the dominant forest types of the area. We found the results correlated with habitat use in a study by Chaiyarat et al. (2019) which indicated that banteng prefer open areas of dry dipterocarp and mixed deciduous forests. This suggests a strong relationship between the availability of preferred forage species and the habitat selection of the banteng. Chaiyarat et al. (2020) analyzed fecal samples collected in 2016 from the same SWS study area, and when compared to our study, they revealed a higher diversity of forage species. The lower diversity we found was likely due to the small population size, and the short time period which had passed since first reintroduction. At that time, only seven captive‐born bantengs had been released, and the study occurred only 1 year after the release (in 2015), so the seven bantengs were still in the process of adapting to their natural habitat. In contrast, our study did monitor at least 8 years after the first release. That is a notable difference, and in that span the population increased naturally to over 30 individuals. This longer period of adaptation and the presence of subsequent generations clearly allowed the banteng population to adjust more effectively to its natural environment. The increased forage diversity found in this study is consistent with studies done on wild banteng populations in other Western Forest Complexes of Thailand. Suksawat et al. (2018) reported 64 plant species consumed by banteng in Huai Kha Khaeng Wildlife Sanctuary (HKK), while Prayurasithi (1987) reported more than 59 forage species in the same study area.

Seasonal differences were found in forage diversity in the feces at SWS, with the higher diversity identified in the wet season. Our finding contradicts results reported by Chaiyarat et al. (2020) which found that forage diversity was lower in the wet season than in the dry season. This may be because of differences in seasons and the microhabitat of sampling areas when the study was conducted.

Banteng in the KKKC consumed a less diverse array of plants than those in SWS across both seasons. The ratio of monocotyledon to dicotyledon was higher during both seasons in the KKKC. The preferred forage species were primarily from the family Poaceae, notably *Panicum maximum* Jacq., *Imperata cylindrica* (L.) Raeusch. and *Brachiaria mutica* (Forssk.) Stapf., which are typically found in areas with open canopies. *Imperata cylindrica* (L.) Raeusch. is also a preferred forage species for Indonesian banteng (Ralls and Halder 1977).

The fecal analysis also identified *Cocos nucifera* L., indicating that the given banteng was foraging in agricultural areas along the forest edge. This suggests a limitation of suitable habitat, as dry evergreen forest—which covers 51.6% of the area—is not a preferred habitat for the species. In both study areas, the identified forage species indicated that banteng primarily forage in open areas and flat habitats. These include mixed deciduous forests, dry dipterocarp forests, secondary forests, and ecotones adjacent to agriculture areas. This finding aligns with previous research.

Phan and Gray (2010) reported that banteng in Cambodia typically avoid evergreen rainforests and prefer more open, dry deciduous forests. Similarly, studies on Javan and Bornean banteng populations have reported a preference for open, dry deciduous forests with occasional use of secondary forests resulting from logging and fires, and are known to enter tracts of sub humid forest on occasion, especially in more humid areas (Wharton 1968). In comparative studies between the two sites, the SWS population exhibited a lower incidence of presence in agricultural areas (Chaiyarat et al. 2019). This was due to the SWS having more available forage species and a larger suitable habitat than KKKC—even though it is not an original banteng habitat.

A seasonal comparison revealed that vegetation diversity was higher during the wet season than the dry season. This observation is likely due to the fact that higher precipitation in the wet season directly influences the growth and variation of plant species available in the area (Chaiyarat et al. 2012). Grasses and other food items are in turn less available in the dry season (Chaiyarat 2002). In other areas, herbivores in the HKK exhibited seasonal differences in diet (McShea et al. 2019). Specifically reported was a higher proportion of woody plants, and a related increased occurrence of specific woody species in the herbivore diet during the dry season—a period when forbs and grasses typically become senescent (i.e., less palatable or nutritious). This seasonal dietary shift is also reported in Africa, where browsers and mixed‐feeders shift to more dicots during the dry season, although grazers generally maintain their monocots diet (Codron et al. 2007). Furthermore, a shift in an animal's food preference during a given season is often linked to modifications in forage palatability. A change in food preference by animals during the vegetative growth season may be linked to the palatability of forage species which correspond to different developmental stages. Various factors—including the texture and chemical composition of the leaves, flavors and odors—can significantly influence animals' preference ratings (Chaiyarat et al. 2021). Herbivore feeding patterns are also influenced by body size and interspecies interactions (Young et al. 2013).

A niche breadth of plant consumption revealed that banteng in SWS are less selective feeders than in KKKC, in both the wet and dry seasons. The difference in foraging diversity largely depends on seasonal effects and habitat quality (Yan et al. 2024). This was especially notable during the dry season, as when only 22 forage species were consumed in KKKC. The SWS, meanwhile, is home to a much broader food niche. This was also due to variations in environmental factors and the vegetation structure of the study areas (Hausharter et al. 2023).

The forage species highly preferred by banteng differ between the two areas and included *Bambusa bambos* (L.) Voss, *Arundinella rupestris* *A. Camus* , *Panicum repens* L., *Bauhinia scandens* L., and *Dendrolobium triangulare* (Retz.) Schindl. – as well as several dicot species found exclusively in fecal samples from SWS, which were not found in banteng feces in KKKC. High consumption frequency among banteng in KKKC included *Imperata cylindrica* (L.) Raeusch., *Panicum maximum* Jacq., and *Brachiaria mutica* (Forssk.) Stapf., all of which were found in feces at KKKC but were not utilized by the banteng population in the SWS. It was evident that SWS contained a greater diversity of forage plants, and this further suggests that SWS provides a more suitable habitat for banteng (Klimm et al. 2024).

Mineral composition analysis has shown that the food plants from both areas contain adequate minerals to support banteng nutritional requirement (National Academies of Sciences, Engineering, and Medicine 2016). The variation in the consumption of forage species by an ungulate may be governed by the amount of a given plant's nutrients. Dostaler et al. (2011) reported a positive relationship between the concentration of crude protein in a plant species and the animal's feeding preference. N is a fundamental component of amino acids. Herbivores require proteins for virtually all bodily functions. Trace minerals such as Cu, Fe, and Zn are essential nutrients for growth, reproduction and normal physiological and immune function in animals (Barboza et al. 2009). In SWS, two dominant plant species contained the highest level of Zn among all species. Zn serves as a vital constituent of numerous enzymes and also acts as an activator for several others. Zinc‐dependent enzymes play key roles in the metabolism of nucleic acids, proteins, and carbohydrates (Hambidge et al. 1986). Moreover, Zn is essential for the normal development and proper functioning of the immune system (National Academies of Sciences, Engineering, and Medicine 2016). The Zn content of forages is affected by a number of factors including plant species, maturity and soil zinc (Minson 1990). This suggests a potential preference by banteng for forage with a specific, high‐mineral nutritional profile. However, animal foraging behavior is also influenced by nutrient food palatability (Yue et al. 2023).

The NMDS results reveal distinct patterns in species' forage composition and mineral content, both between the two study areas and across seasons, suggesting that both geographic and temporal factors play significant roles in shaping the nutritional landscape available to herbivores in these regions. These groupings suggest that seasonal variation plays an important role in shaping the nutritional profiles of selected forage species. However, banteng also obtain mineral supplements directly from saltlicks. Saltlicks are an important mineral source for wildlife, especially herbivores (Razali et al. 2020). These findings highlight the importance of seasonal foraging strategies and habitat‐specific plant nutrient profiles in shaping herbivore diet selection.

The difference in population growth between the two sites reflects the influence of habitat suitability, release strategy, and pre‐release preparation on the success of reintroduction programs. Soft‐release strategies are generally more effective than hard‐release approaches for captive and terrestrial species, as providing an acclimatization period prior to release helps reduce stress‐related issues (Teixeira et al. 2007; Resende et al. 2021). This situation differs markedly from the well‐planned reintroduction in the SWS, where both ecological and social factors were systematically considered. The contrast between these two cases underscores the importance of comprehensive planning, habitat assessment, and adherence to established reintroduction guidelines in ensuring the long‐term stability of reintroduced wildlife populations.

## 5 Conclusion

The successful establishment of a banteng population, particularly following reintroduction, depends on species‐specific ecological requirements. Understanding banteng foraging behavior and dietary nutrient quality is essential for habitat management. This study found that mineral compositions in natural forage species provide adequate minerals to support banteng nutritional requirements. This result indicates that the long‐term survival of banteng after reintroduction depends on a suitable habitat. Protection of forages that provide quality nutrition can support the reintroduced population; thus, habitat improvements are required to ensure the long‐term sustainability of the banteng population.

## Author Contributions

**Wasinee Thepapichaikul:** data curation (equal), formal analysis (equal), investigation (equal), methodology (equal), software (equal), visualization (equal), writing – original draft (equal). **Rattanawat Chaiyarat:** conceptualization (equal), data curation (equal), formal analysis (equal), funding acquisition (equal), investigation (equal), methodology (equal), project administration (equal), resources (equal), software (equal), supervision (equal), validation (equal), writing – review and editing (equal). **Roschong Boonyarittichaikij:** conceptualization (equal), methodology (equal), supervision (equal), validation (equal). **Phanwimol Tanhan:** conceptualization (equal), supervision (equal), validation (equal). **Seree Nakbun:** data curation (equal), validation (equal).

## Funding

This work was supported by Mahidol University (Fundamental Fund: fiscal year 2023 by National Science Research and Innovation Fund [NSRF]), FF‐116/2566.

## Conflicts of Interest

The authors declare no conflicts of interest.

## Supporting information


**Table S1:** The difference in details between two banteng reintroduction area: Khao Kiew–Khao Chompoo Wildlife Sanctuary (KKKC) and Salakphra Wildlife Sanctuary (SWS), Thailand.
**Table S2:** The nutrition contents in forage species between two banteng reintroduction area: Khao Kiew–Khao Chompoo Wildlife Sanctuary (KKKC) and Salakphra Wildlife Sanctuary (SWS), Thailand.

## Data Availability

The original data are contained within the article and Tables S1 and S2.
