# Supplementary material for: Forage Species and Nutrition Among Reintroduced Banteng (Bos javanicus d'Alton, 1823) in Salakphra Wildlife Sanctuary and Khao Kiew–Khao Chompoo Wildlife Sanctuary, Thailand
Source: Ecol Evol. 2026 Apr 1;16(4):e73401. doi: 10.1002/ece3.73401 (PMC13045356; doi:10.1002/ece3.73401)
Supplement: Supplementary file 1 — Table S1: The difference in details between two banteng reintroduction area: Khao Kiew–Khao Chompoo Wildlife Sanctuary (KKKC) and Salakphra Wildlife Sanctuary (SWS), Thailand. Table S2: The nutrition contents in forage species between two banteng reintroduction area: Khao Kiew–Khao Chompoo Wildlife Sanctuary (KKKC) and Salakphra Wildlife Sanctuary (SWS), Thailand. [file ECE3-16-e73401-s001.docx]

**SUPPLEMENTSRY TABLE 1.** The difference in details between two banteng reintroduction area: Khao Kiew–Khao Chompoo Wildlife Sanctuary (KKKC) and Salakphra Wildlife Sanctuary (SWS), Thailand.

| **Factors** | **KKKC** | **SWS** |
| --- | --- | --- |
| Province | Chonburi | Kanchanaburi |
| Area | 114.7 km^2^ | 860 km^2^ |
| Land cover | 51.6% dry evergreen forest, 37.5% mixed deciduous forest, 6.3% agricultural area, 2.1% grassland, 2.2% secondary forest 0.3% water sources | 60% mixed deciduous forest  30% dry dipterocarp forest  10% disturbed areas |
| Releasing type by definition | Introduction | Reintroduction |
| Releasing approach | Hard release | Soft release |
| Year | 1988 | 2015-2022 (5 events) |
| Number of banteng released | 13 individuals | 16 individuals |
| Current population | 30-40 individuals | >60 individuals |
| Post-monitoring | Chaiyarat et al., (2018) | Chaiyarat et al., (2020) |

**SUPPLEMENTARY TABLE 2.** The nutrition contents in forage species between two banteng reintroduction area: Khao Kiew–Khao Chompoo Wildlife Sanctuary (KKKC) and Salakphra Wildlife Sanctuary (SWS), Thailand

| Species | Area | Season | Nutrition (%) | | | | | | (Mg/kg) | | | | |
| --- | --- | --- | --- | --- | --- | --- | --- | --- | --- | --- | --- | --- | --- |
|  |  |  | N | P | S | K | Ca | Mg | Na | Cu | Fe | Mn | Zn |
| Pm | KKKC | Dry | 2 | 0.14 | 1.3 | 1.8 | 0.2 | 0.1 | 139.9 | 3.7 | 86.4 | 63.3 | 34.5 |
| Ic | KKKC | Dry | 1.1 | 0.2 | 1 | 0.6 | 0.4 | 0.1 | 89.5 | 3.3 | 108.9 | 59.4 | 19.7 |
| Bm | KKKC | Dry | 2.3 | 0.2 | 2.9 | 1.9 | 0.4 | 0.2 | 1,849.2 | 5.9 | 88 | 32.6 | 40.8 |
| Ss | KKKC | Dry | 0.8 | 0.2 | 0.9 | 0.9 | 0.2 | 0.1 | 73.3 | 2.6 | 44.4 | 71.5 | 6.8 |
| Sac | KKKC | Dry | 1.5 | 0.2 | 1.2 | 1 | 0.2 | 0.1 | 58.4 | 0.8 | 36.4 | 63.1 | 13.4 |
| Ts | KKKC | Dry | 3 | 0.1 | 1.9 | 0.9 | 0.5 | 0.3 | 97.9 | 3.1 | 138.3 | 127.5 | 33.1 |
| Sar | KKKC | Dry | 1.6 | 0.2 | 2.3 | 1.3 | 0.1 | 0.1 | 79 | 7.6 | 35.8 | 32.6 | 21 |
| Cnu | KKKC | Dry | 1.1 | 0.2 | 1.2 | 1.7 | 0.1 | 0.2 | 2,140.6 | 7 | 42.4 | 55.7 | 14.3 |
| Dh | KKKC | Dry | 4.5 | 0.4 | 2.8 | 1.2 | 1.2 | 0.3 | 103.1 | 5.8 | 441.7 | 120.5 | 30.7 |
| UnkDi13 | KKKC | Dry | 3 | 0.2 | 1.7 | 1.3 | 0.6 | 0.2 | 69.1 | 10.6 | 19.6 | 78.1 | 28.9 |
| Pm | KKKC | Wet | 1.5 | 0.2 | 1.2 | 1.1 | 0.3 | 0.2 | 21.5 | 20.8 | 49.8 | 36 | 21.2 |
| Ic | KKKC | Wet | 1.1 | 0.1 | 1 | 0.4 | 0.5 | 0.1 | 7.3 | 12 | 46.4 | 132.2 | 16.7 |
| Bm | KKKC | Wet | 2.1 | 0.3 | 2 | 1.8 | 0.3 | 0.2 | 1,121.5 | 10.5 | 183.6 | 142.7 | 31.8 |
| Ss | KKKC | Wet | 1.4 | 0.2 | 1.1 | 0.4 | 1.1 | 0.1 | 45.2 | 10.1 | 95.2 | 39.2 | 22.6 |
| Op | KKKC | Wet | 1.8 | 0.4 | 2.2 | 2 | 0.4 | 0.2 | 51.1 | 11.3 | 121.7 | 30.6 | 21.7 |
| Sac | KKKC | Wet | 1.4 | 0.1 | 1.1 | 0.8 | 0.4 | 0.1 | 13.4 | 10.6 | 85.2 | 82.6 | 17.7 |
| Hr | KKKC | Wet | 0.7 | 0.1 | 1.1 | 1 | 0.6 | 0.1 | 38.1 | 8.7 | 147.7 | 65.8 | 34 |
| Sas | KKKC | Wet | 1.7 | 0.2 | 0.6 | 1.1 | 7.1 | 0.6 | 17 | 15.2 | 74 | 708.4 | 66.7 |
| Cba | KKKC | Wet | 1.6 | 0.4 | 3 | 1.3 | 0.2 | 0.1 | 2,453.1 | 10.7 | 117.2 | 61.9 | 35.7 |
| Ch | KKKC | Wet | 1.9 | 0.2 | 0.8 | 2.9 | 0.8 | 0.2 | 28.2 | 12.5 | 256.1 | 188.9 | 44.3 |
| Cy | SLP | Dry | 1.6 | 0.2 | 2.5 | 1.6 | 0.6 | 0.4 | 47.1 | 19.5 | 421.9 | 83.9 | 42.5 |
| Bp | SLP | Dry | 3.1 | 0.2 | 2.7 | 1.7 | 0.2 | 0.1 | 138.6 | 5.7 | 150.5 | 66.2 | 53.8 |
| Bsc | SLP | Dry | 2 | 0.2 | 1 | 1.2 | 1.5 | 0.3 | 87.2 | 5 | 52.3 | 32.7 | 4.6 |
| Ts | SLP | Dry | 2.3 | 0.1 | 1.6 | 1.1 | 0.1 | 0.1 | 74 | 2.4 | 96.3 | 33.1 | 10.6 |
| Amu | SLP | Dry | 0.8 | 0.1 | 1.2 | 0.8 | 0.3 | 0.2 | 62.9 | 14.7 | 249.1 | 151.3 | 28.8 |
| Bsa | SLP | Dry | 3.1 | 0.3 | 1.7 | 1.2 | 1 | 0.3 | 59.3 | 13.9 | 103.6 | 51.7 | 35 |
| Zo | SLP | Dry | 3.2 | 0.2 | 1.5 | 1.3 | 2.2 | 0.6 | 55.2 | 6.1 | 96.8 | 1,356.8 | 41.7 |
| Mb | SLP | Dry | 3.3 | 0.2 | 1.5 | 1.3 | 0.8 | 0.2 | 58.4 | 13.1 | 84 | 21.8 | 29.1 |
| Dcu | SLP | Dry | 2.4 | 0.2 | 1.6 | 1.3 | 2.5 | 0.4 | 73.2 | 1.2 | 54.5 | 149.5 | 4.1 |
| Cs | SLP | Dry | 2.9 | 0.3 | 1.1 | 1 | 0.1 | 0.1 | 56 | 16.1 | 73.9 | 29.8 | 28.6 |
| Cy | SLP | Wet | 0.9 | 0.2 | 1.4 | 2 | 0.3 | 0.2 | 37.4 | 17.2 | 1,043.9 | 113.7 | 36.4 |
| Dt | SLP | Wet | 2.5 | 0.2 | 2.4 | 1.1 | 1.3 | 0.4 | 21.1 | 23.8 | 88.1 | 128.9 | 46 |
| Ds | SLP | Wet | 1.8 | 0.2 | 1.4 | 0.9 | 1.5 | 0.4 | 21.8 | 15.6 | 134.2 | 229.8 | 35.8 |
| Cba | SLP | Wet | 1.3 | 0.2 | 1.9 | 1.3 | 0.7 | 0.2 | 21.9 | 19.2 | 173.7 | 33.4 | 37.7 |
| Ar | SLP | Wet | 0.8 | 0.1 | 1.6 | 0.8 | 0.1 | 0.2 | 41.2 | 17.3 | 336.9 | 150.7 | 24.5 |
| Prep | SLP | Wet | 0.9 | 0.2 | 1.9 | 1.2 | 0.4 | 0.1 | 28.6 | 17.6 | 418.7 | 72.7 | 30.6 |
| Bb | SLP | Wet | 2.1 | 0.2 | 3.4 | 1.2 | 0.3 | 0.2 | 39.1 | 24.8 | 142.7 | 51.5 | 45.9 |
| Dci | SLP | Wet | 1.1 | 0.2 | 2.9 | 1.3 | 0.5 | 0.2 | 25.8 | 20.3 | 154.6 | 113.5 | 25.9 |
| Fi | SLP | Wet | 0.5 | 0.1 | 1.3 | 0.9 | 0.2 | 0.2 | 32.4 | 21 | 2,725.1 | 303.2 | 33.9 |
| Bsc | SLP | Wet | 2.8 | 0.1 | 2.3 | 0.9 | 4.9 | 0.5 | 53.3 | 27.5 | 151.5 | 32.7 | 28 |

Note: KKKC = Khao Kiew – Khao Chompoo Wildlife Sanctuary, SWS = Salakphra Wildlife Sanctuary, Amu = *Apluda mutica* L., Ar = *Arundinella rupestris* A.Camus*,* Bb = *Bambusa bambos* (L.) Voss, Bm = *Brachiaria mutica* (Forssk.) Stapf, Bsa = *Bauhinia saccocalyx* Pierre, Bsc = *Bauhinia scandens* L., Cba = *Chloris barbata* Sw., Cnu = *Cocos nucifera* L., Ch = *Cyperus haspen* L., Cs = *Caesalpinia sappan* L., Cy = *Cyrtococcum* sp., Dci = *Digitaria ciliaris* (Retz.) Koeler. var. *chrysoblephara* (Figari & De Notaris) R. R. Stewart, Dcu = *Dalbergia cultrata* Graham ex Benth., Dh = *Dioscorea hispida* Dennst., Ds = *Dendrocalamus strictus* (Roxb.) Nees*,* Dt = *Dendrolobium triangulare* (Retz.) Schindl., Fi = *Fimbristylis insignis* Thwaites., Hr = *Hyparrhenia rufa* (Nees) Stapf*,* Ic = *Imperata cylindrica* (L.) Raeusch., Mb = *Millettia brandisiana* Kurz, Op = *Ophiuros* sp., Pm = *Panicum maximum* Jacq., Prep = *Panicum repens* L., Sac = *Saccharum* sp., Sas = *Streblus asper* Lour., Sar = *Saccharum arundinaceum* Retz., Ss = *Saccharum spontaneum* L.*,* Ts = *Thyrsostachys siamensis* Gamble, UnkDi13 = Unknow dicotyledon species number 13, Zo = *Ziziphus oenoplia* (L.) Mill. var. *oenoplia*.
